# Sustainable optimization of high specific surface area *Spartina alterniflora* biochar for Rhodamine B removal and mechanism

**DOI:** 10.1038/s41598-025-05714-6

**Published:** 2025-07-01

**Authors:** Weiling Yu, Zhouyun Xie, Ni Zhang, Li Tang, Jingfen Xia, Jien Ye, Xuran Liu, Dongbo Wang, Guojing Yang

**Affiliations:** 1 College of Biological and Environmental Sciences, Zhejiang Wanli University, Ningbo, 315100 People’s Republic of China; 2 College of Environmental Science and Engineering, Hunan University and Key Laboratory of Environmental Biology and Pollution Control (Hunan University), Ministry of Education, Changsha, 410082 People’s Republic of China

**Keywords:** *Spartina alterniflora*, KOH activation, Biochar, Rhodamine B, Adsorption mechanism, Pollution remediation, Environmental impact

## Abstract

Managing dye contaminants is a major challenge in modern water governance. This study developed a KOH-activated *Spartina alterniflora* biochar (KBC) adsorbent for removing Rhodamine B (RhB) dye from water. Microscopic analysis validated the presence of an appealing porous structure and surface functionalities that are key for the adsorption of RhB. KBC demonstrated an enormous specific surface area, providing many active sites (3109.67 m^2^·g^−1^). Optimal circumstances for eliminating RhB were achieved an impressive 89.77% at a pH level of 7, utilizing a KBC dosage of 100 mg/100 mL over a contact period of 48 h, resulting in a maximum adsorption capacity of 1820.47 mg·g^−1^. Furthermore, according to the findings, pseudo-second-order, Langmuir, and Freundlich models offered a precise match of the batch experiment results. The combined effects of the KBC pore filling, π–π electron donor–acceptor (π–π EDA), hydrogen bonding, and electrostatic interactions facilitated both physical and chemical adsorption mechanisms, which in turn bolstered the biochar’s superior adsorption capabilities. These results underscore the viability of KBC as a promising candidate for water purification, demonstrating its potential to eliminate RhB from polluted waters for sustainable cleanup efforts effectively and the resource utilization of waste.

**Supplementary Information:**

The online version contains supplementary material available at 10.1038/s41598-025-05714-6.

## Introduction

Organic dyes are widely recognized as emerging contaminants and are extensively used in the textile, plastic, and leather industries. Globally, over 70 million tons of synthetic dyes are produced each year, contributing to approximately 70% of the total industrial wastewater, which severely contaminates surface water bodies^1^. Rhodamine B (RhB), a synthetic triphenylmethane dye known for its structural stability, is broadly applied in acrylic fiber dyeing, leather tanning, papermaking, and as a food additive^2,3^. Owing to its molecular structure, which contains functional groups such as benzene rings, carboxyl, and amino groups, RhB exhibits high environmental stability and strong resistance to biodegradation, making it a representative refractory organic pollutant^4^. Once released into aquatic ecosystems, RhB not only reduces light penetration but also interferes with natural purification processes, thereby posing risks to both ecological communities and human health^5^. To date, a variety of technologies have been employed for dye removal from wastewater, including adsorption, electrochemical degradation, coagulation-flocculation, membrane filtration, and biological treatment. While these methods can achieve satisfactory removal efficiency under specific conditions, they often face limitations such as high operational costs, substantial energy consumption, complex equipment requirements, and the potential for secondary pollution- especially when treating high concentrations of organic dyes^6–8^. Among them, adsorption has received considerable attention due to its operational simplicity, broad applicability to different dye types, and the absence of toxic by-products. Therefore, the development of adsorbents with high adsorption capacity, low production cost, and strong environmental sustainability is of great importance for advancing efficient and eco-friendly wastewater treatment strategies.

Biochar is a cost-effective and environmentally sustainable material produced by pyrolyzing biomass under oxygen-limited or anaerobic conditions^9,10^. Its low production cost, stable physicochemical properties, and abundant functional groups make it widely applicable for the adsorption of various environmental pollutants^11,12^. Biochar can be derived from a wide range of feedstocks, and its physicochemical properties and surface structures vary significantly depending on the raw materials used^13,14^. Consequently, its adsorption performance is closely linked to the biomass source. *Spartina alterniflora*, a perennial salt-tolerant herb, has rapidly spread since its introduction to China, displacing native plant species and forming monocultures that disrupt ecosystem structure and function, ultimately reducing regional biodiversity^15,16^. Various physical, chemical, and biological control methods have been employed to manage its spread. However, these methods require substantial labor and resources and may not provide an optimal long-term solution. Due to its high carbon content (47.3%), *Spartina alterniflora* has been identified as a promising feedstock for biochar production^17^. Li et al. successfully converted *Spartina alterniflora* into biochar and demonstrated its effectiveness as an adsorbent for copper (Cu^2+^) removal^18^. Therefore, biochar derived from *Spartina alterniflora* represents a viable high-value utilization strategy that not only mitigates its invasive spread but also promotes waste recycling, contributing to a sustainable “waste-to-resource” approach.

However, biochar derived from *Spartina alterniflora* and other biomass feedstocks typically exhibits a limited specific surface area and poorly developed pore structures, which constrain its adsorption capacity. To address this limitation, it is crucial to develop effective activation and modification strategies to further enhance the adsorption performance of *Spartina alterniflora* biochar. Among various approaches, chemical activation is generally regarded as more effective than physical activation, with alkali activation being a particularly efficient method for significantly increasing the specific surface area and porosity of biochar^19^. Commonly used alkali activators include KOH and NaOH, with KOH demonstrating superior intercalation capability and catalytic properties, making it more effective than NaOH in enhancing pore development^20^. Compared with one-step activation, the two-step activation process not only increases the specific surface area but also improves the biochar yield. Qu et al. converted corn stalks into highly porous biochar using a two-step KOH carbonization process, achieving a specific surface area of 2183.80 m^2^·g^−1^^21^.

Based on these considerations, this study proposes the KOH activation of *Spartina alterniflora* biochar to fabricate a porous carbonaceous material and evaluates its effectiveness in removing RhB from aqueous solutions. A series of characterization techniques is employed to analyze the structural properties of *Spartina alterniflora* biochar before and after activation. Additionally, its adsorption behavior under controlled experimental conditions is investigated to elucidate the underlying mechanisms of RhB removal. By converting *Spartina alterniflora* into a biochar-based adsorbent, this study offers a sustainable and practical approach to improving waste management, mitigating environmental pollution, and promoting the efficient utilization of resources.

## Materials and methods

### Materials and reagents

The *Spartina alterniflora* for the experiment was obtained from Beilun, Ningbo. The *Spartina alterniflora* was washed, air-dried, pulverized into a fine powder using a pulverizer, and sieved to obtain a 60-mesh size. Subsequently, it was preserved in a transparent desiccator.

Potassium hydroxide (KOH, > 85%), sodium hydroxide (NaOH, > 96%), hydrochloric acid (HCl, 36.0%–38.0%), and Rhodamine B (RhB, C_28_H_31_ClN_2_O_3,_ > 98%) were acquired from Sinopharm Chemical Reagent Co., Ltd, all reagents used were of analytical grade. Ultrapure water was obtained from the laboratory.

### Preparation and modification of biochar

A specified quantity of *Spartina alterniflora* powder was placed in a quartz boat and subsequently loaded into the chamber of a tube furnace (OTF-1200X-IV-S, China). Pyrolysis was conducted in a nitrogen atmosphere at 450 °C for 2 h, with the heating rate set at 10 ℃·min^−1^ and the N_2_ atmosphere rate at 1 L·min^−1^. After the process, the resulting product was allowed to cool naturally and ground to pass through a 100-mesh sieve. The finely ground material was stored in a glass desiccator and designated as BC450.

The BC450 material obtained from the prior phase was mixed with KOH powder (> 85% purity) at a solid-state mass ratio of 1: 2.5 (BC450: KOH activator = 1 g: 2.5 g)^22^. The resulting mixture was transferred into a nickel vessel and subjected to pyrolysis in a tubular furnace. Activation was performed at different temperatures (600, 700, 800, and 900 °C) under an N_2_ atmosphere rate at 1 L·min^−1^, with the heating time and rate maintained as described above. After natural cooling, the sample was rinsed with HCl (1 M) to remove residual KOH, followed by leaching with ultrapure water to achieve neutralization. After drying, the specimens were filtered through a 100-mesh sieve. The finely ground material was stored in a glass desiccator and labeled as KBC600, KBC700, KBC800, and KBC900.

### Characterization of biochar

The morphological features and chemical components of the sample were examined via an array of analytical techniques, as outlined in Text S1.

### Batch adsorption experiment

The RhB compound was completely dissolved in ultrapure water for the preparation of a 10 g·L^−1^ stock solution. Weighing out 0.1 g of BC and KBC, respectively, into a 250 mL conical flask, they are combined with 100 mL of RhB solution at a concentration of 2000 mg·L^−1^. All adsorption experiments were carried out in a thermostatic shaker maintained at 25 °C and 150 rpm. Following centrifugation, the supernatant was collected for further analysis. To investigate the effect of pH, 0.1 g of biochar was mixed with either 0.1 M HCl or 0.1 M NaOH to adjust the pH range from 3 to 11. The influence of biochar dosage on adsorption was examined by varying the biochar amount from 0.1 to 0.25 g. The impact of temperature and thermodynamic parameters was evaluated at 25, 30, and 35 °C. Kinetic studies were conducted with reaction times ranging from 0 to 48 h. Adsorption isotherms were analyzed using RhB concentrations ranging from 100 to 4000 mg·L^−1^. Every experiment is conducted in triplicate. The adsorption capacity and RhB removal efficiency were calculated using Eqs. (1)–(2), respectively.

1 $${\text{q}}_{{\text{e}}} { = }\frac{{{\text{(C}}_{{0}} {\text{ {-} C}}_{{\text{e}}} {\text{)V}}}}{{\text{m}}},$$

2 $$\eta { = }\frac{{{\text{(C}}_{{0}} {\text{ {-} C}}_{{\text{e}}} {)}}}{{{\text{C}}_{{0}} }} \times 100\% ,$$

where the initial concentration of the RhB solution is *C*_0_ (mg·L^−1^), *C*_e_ represents the equilibrium mass concentration of the RhB solution, expressed in mg·L^−1^, *V* denotes the RhB solution volume (L), *m* indicates the adsorbent’s mass (g), $\eta$ stands for removal rate (%).

### Data processing and analysis

An ultraviolet spectrophotometer (UV, AOE Instrument 1800, China) was used to determine the removal efficiency of RhB at 554 nm. The details of adsorption kinetics, isotherms, and thermodynamics are supplied in Table S2. Each experiment was performed three times for the sake of replicability.

## Results and discussion

### Adsorption performance evaluation

The influence of BC and KBC on RhB adsorption at varying pyrolysis temperatures is illustrated in Fig. S1. Adsorption amounts of RhB with KBC600, KBC700, KBC800, and KBC900 were 1323.22, 1721.74, 1820.47, and 1850.65 mg·g^−1^, respectively, whereas the quantity for BC450 was only 23.42 mg·g^−1^. The activation process using KOH markedly enhances BC’s adsorption capability and efficacy in removal. This was due to the activation temperature of 600 °C, KOH will react with C to generate K_2_CO_3_. As the activation temperature continues to rise to 700 °C and above, K_2_CO_3_ decomposes into K_2_O and CO_2_, forming a large number of pore structures, and increasing the adsorption sites^23^. The pyrolysis process occurs in the chemical reaction as shown in the following reaction equations (Eqs. (3)–(7)). However, when the activation temperature increased to 900 °C, the interaction between KBC and KOH became highly vigorous, leading to the collapse of some micropores. This disruption in the pore structure compromised its stability, and as a result, the adsorption capacity did not show substantial enhancement. Thus, for this study, KBC800 was chosen as the sorbent and BC450 served as the control to examine the sorption properties and mechanism of RhB onto unmodified and modified biochar.

When the activation temperature is 600 °C^21,24^

3 $${\text{2C }} + {\text{ 6KOH}} \to {\text{2K }} + {\text{ 3H}}_{{2}} + {\text{ 2K}}_{{2}} {\text{CO}}_{{3}}$$

When the activation temperature is > 700 °C^25^

4 $${\text{K}}_{{2}} {\text{CO}}_{{3}} \to {\text{K}}_{{2}} {\text{O }} + {\text{ CO}}_{{2}}$$

5 $${\text{K}}_{{2}} {\text{CO}}_{{3}} + {\text{ CO}}_{{2}} \to {\text{K}}_{{2}} {\text{O }} + {\text{ 2CO}}$$

6 $${\text{C }} + {\text{ K}}_{{2}} {\text{O}} \to {\text{2K }} + {\text{ CO}}$$

7 $${\text{CO}}_{{2}} + {\text{ C}} \to {\text{2CO}}$$

### Structural and morphological analysis

**Fig. 1:**
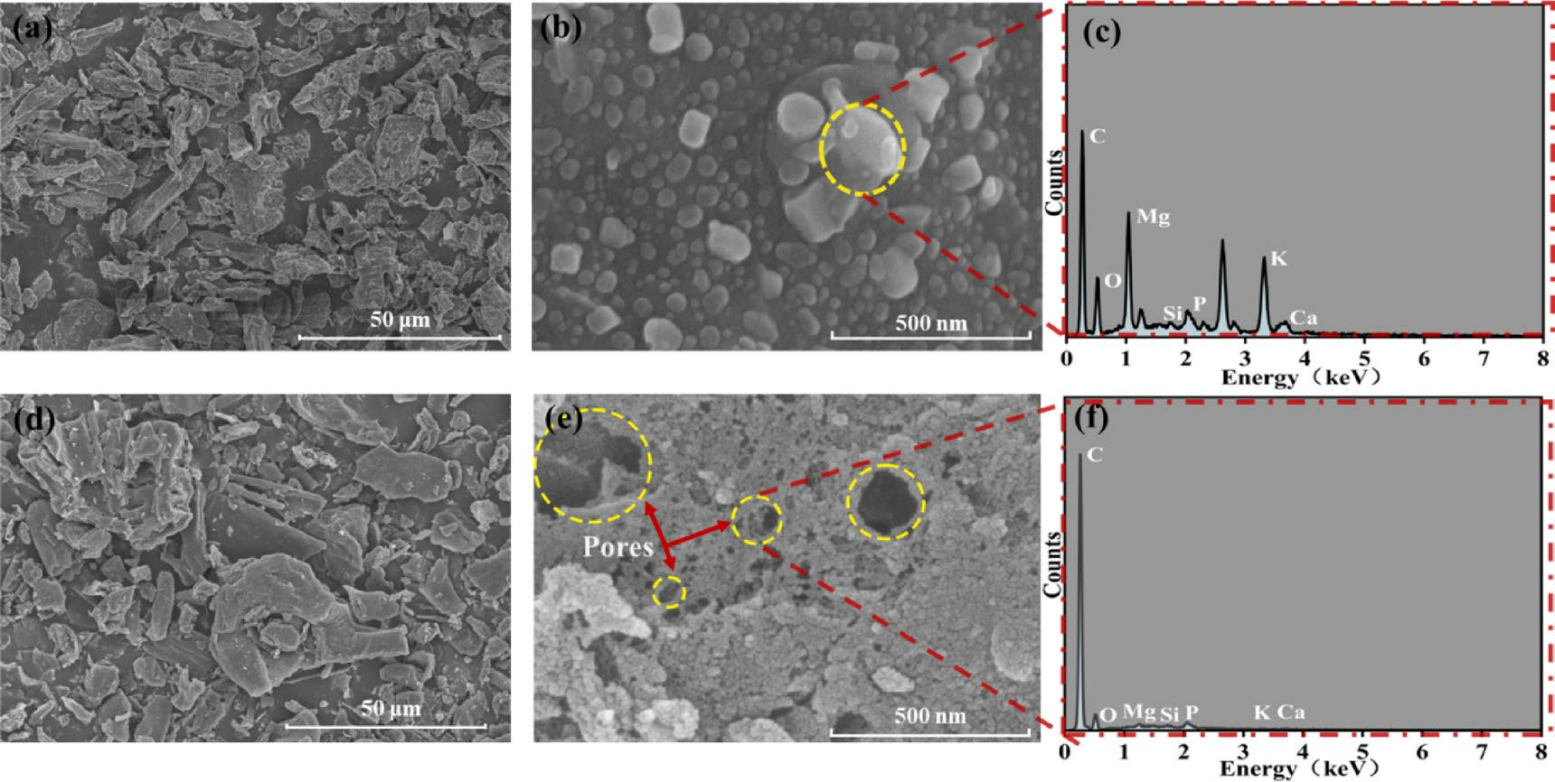
(**a**, **b**) SEM images of BC, (**c**) EDS of BC, (**d**, **e**) SEM images of KBC and (**f**) EDS of KBC.

Samples were analyzed using a scanning electron microscope (SEM) to examine their images. Comparative analysis of Fig. 1 revealed that the surface of the unactivated BC was a compact lamellar stacking arrangement with poorly defined pores (Fig. 1a, b), indicating limited pore formation during pyrolysis carbonization at 450 °C, whereas that of the KBC was etched due to KOH at high temperature, which favored developing irregular and uneven pores (Fig. 1d, e)^23,26^. The porous structure of KBC provided more adsorption sites for RhB, and its diffusion into the interior of KBC enhanced its adsorption performance.

X-ray energy dispersive spectroscopy (EDS) (Fig. 1c) indicated the detection of elements C, O, N, Mg, K, and P, which are commonly found in BC due to the raw material. After activation, KBC had a high proportion of C, the ash components (K, Ca, Mg, etc.) were reduced, and simultaneously the interaction of KBC with oxygen-bearing functional moieties during pyrolysis led to the elimination of the majority of oxygen-containing substituents (Fig. 1f). The process generated many vacancies and formed a new pore structure. KOH activation effectively enhanced the SSA and porosity of biochar, as displayed by the distribution of O content.

**Fig. 2:**
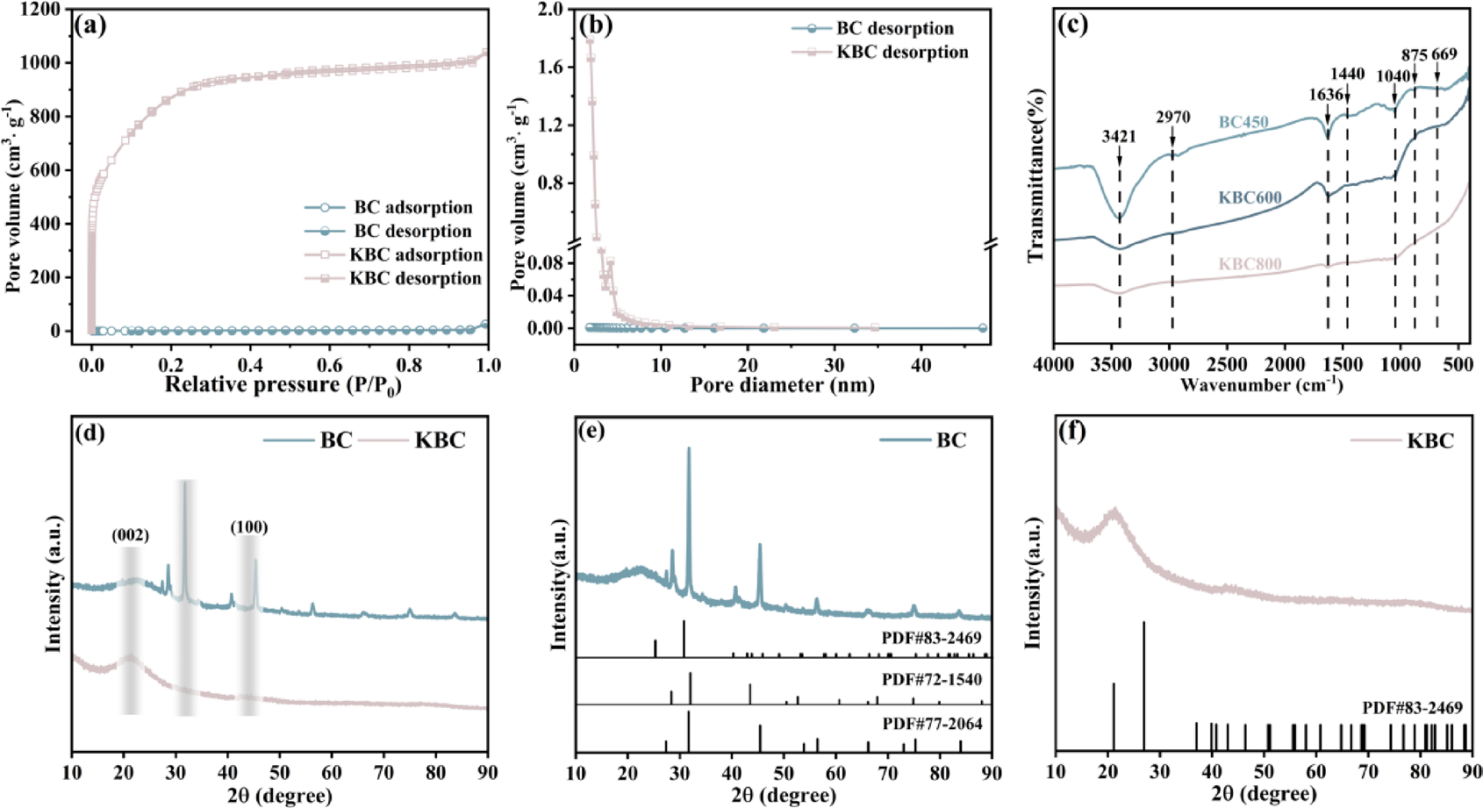
(**a**) N_2_ adsorption–desorption curves of BC and KBC and (**b**) pore size distribution, (**c**) FT-IR spectra of BC and KBC, (**d**) XRD patterns of (**e**) BC and (**f**) KBC.

Figure 2a shows the N_2_ adsorption–desorption isotherms and pore size distribution profiles of the samples. It was evident that the adsorption–desorption curves for both BC and KBC conformed to the IUPAC class I phase. The BC curves displayed no significant hysteresis loop or overlap, indicating a relatively uniform mesopore distribution and volume within BC. By contrast, the KBC adsorption curves exhibited a rapid increase at *p*/*p*_0_ < 0.1, suggesting an abundance of microporous structures. At *p*/*p*_0_ > 0.4, a desorption hysteresis phenomenon became apparent, forming an H4-type hysteresis loop, which indicated the presence of mesopores in KBC^27^. Furthermore, when *p*/*p*_0_ > 1, the curve continued to trend upward, signifying the existence of trace macropores.

Table 1 summarizes the SSA and pore volume characteristics of BC and KBC. The SSA of KBC was found to be approximately 554.22 times higher than that of BC, indicating a significant increase resulting from KOH activation. As shown in Fig. 2b, BC exhibited a notably small pore volume at merely 0.04 cm^3^·g^−1^, with most surface pores falling within the 2–5 nm range and an average pore diameter of 28.81 nm. In contrast, the KOH activation modified the pore size distribution, it shifted to a range of 2–10 nm, featuring prominent peaks between 3 and 5 nm. The mean pore diameter reduced to 2.07 nm, while the overall pore volume dramatically to 1.61 cm^3^·g^−1^, representing a 40.25-fold increase compared to BC. This substantial enhancement in pore volume was attributed to the release of gases, such as CO, CO_2_, H_2_O, and H_2_, during the activation process, which promoted pore formation. Furthermore, the incorporation of metallic potassium into the carbon matrix and the subsequent expansion of the carbon lattice were found to significantly enhance the overall porosity structure. These results highlight the beneficial effects of KOH activation on both specific surface area and porous characteristics in biochar^28^.

**Table 1:** Pore structure parameters of BC and KBC.

Material	*S*_BET_/m^2^·g^−1^	*V*_t_/cm^3^·g^−1^	*S*_mic_/cm^3^·g^−1^	*d*_*p*_/nm
BC	5.60	0.04	0.0017	28.81
KBC	3109.67	1.61	1.42	2.07

Fourier transform infrared spectrometer (FT-IR) analysis was conducted to identify differences in the chemical groups within BC and KBC, as depicted in Fig. 2c. BC exhibited an –OH bond stretching vibration peak near 3450 cm^−1^, attributed to alcohol or phenols. Peaks around 2970 cm^−1^ likely represented methyl (–CH_3_) or methylene (–CH_2_). The intensity at 1636 cm^−1^ signified the stretching of C=C/C=O vibrations, whereas the aliphatic –CH stretching peak was around 1440 cm^−1^. Characteristics of C–O or Si–O–Si stretches were observed at approximately 1040 cm^−1^, while the peaks corresponding to C=O and C–O vibrations were situated around 875 cm^−1^. Different from BC, KOH reacted with carbon to disrupt the structure of cellulose within the original material, resulting in the volatilization of functional groups^29^, the depletion of organic matter, and the cleavage of –OH and hydrogen bonds^30^. This process subsequently weakened or even eliminated the peak positions linked to OH, C=C/C=O, C–O, and aromatic –CH groups in KBC. Consequently, while increasing the SSA, KBC simultaneously diminished the availability of functional group sites for surface complexation, which aligns with Wang et al. findings^31^. The consistent presence of the Si–O absorption peak in biochar was a significant observation^32^, which corroborated observations made through X-ray diffractometry (XRD).

XRD patterns revealed that the composition of BC was primarily composed of SiO_2_ (PDF#83-2469), NaCl (PDF#77–2064), and KCl (PDF#72-1540) (Fig. 2e), while KBC consisted of SiO_2_ (PDF#83-2469) (Fig. 2f). Notably, as shown in Fig. 2d, corresponding to the (002) crystallographic plane of graphite. This feature is commonly attributed to the overlap between the broad amorphous peak of SiO_2_ and the (002) reflection of graphitic carbon, which masks the diffraction signal of crystalline SiO_2_^24,33^. KOH activation significantly affects the carbon structure, and its corrosiveness will destroy the order of the carbon layer, degrade the crystallinity, widen the (002) peak and enhance the dispersion characteristics^34^. A peak around 34° was associated with the formation of elemental Si^35^. In the presence of KOH, Si underwent partial melting, which increased structural disorder and led to a flattened diffraction signal in this region. Additionally, the (100) diffraction plane of graphite was observed at approximately 43.3°, indicating the presence of randomly oriented small graphite fragments in KBC^36^. These fragments are considered π-electron donors and may contribute to the material’s enhanced adsorption properties^37^.

**Fig. 3:**
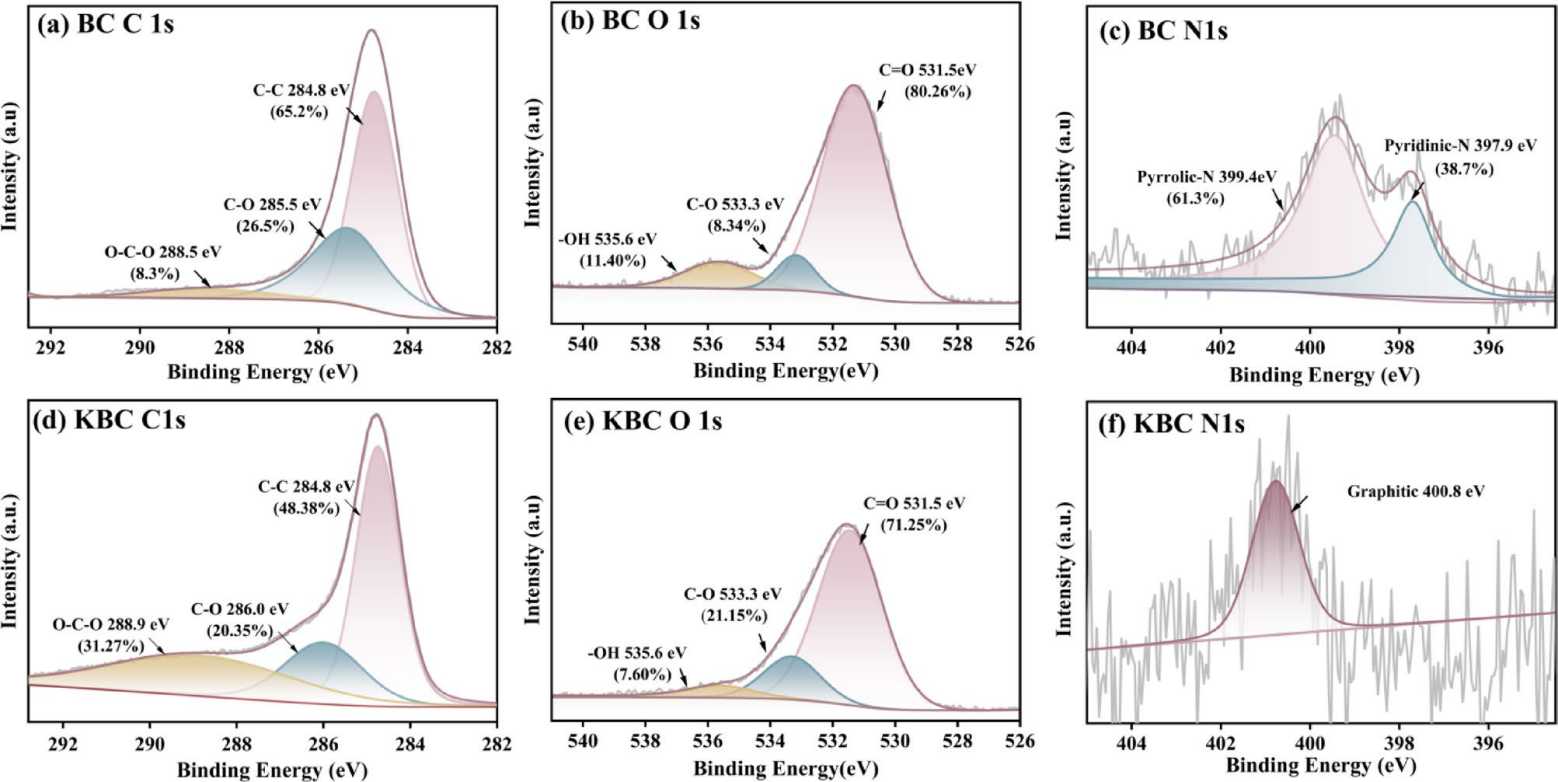
(**a**, **d**) XPS C 1 s, (**b**, **e**) O 1 s, and (**c**, **f**) N 1 s spectra of BC and KBC.

X-ray photoelectron spectrometer (XPS) analysis was conducted to examine the surface composition and identify chemical functionalities of the specimens, with results illustrated in Fig. 3. The C 1 s spectra for both BC and KBC showed three peaks of varying intensities (Fig. 3a, d), corresponding to C–C, C–O, and O–C–O. Among these, C–C represented the predominant component of biochar, which was emphasized by the graphite structure observed in both BC and KBC, aligning with findings from XRD. In comparison to BC, the proportion of C–O in KBC was significantly changed due to some C–C being converted into C–O. This alteration suggested that BC and KBC surface groups were more likely to engage in hydrogen bonding with RhB^38^. The O 1 s spectra reveal distinct peaks at 531.5 eV, 533.3 eV, and 535.6 eV (Fig. 3b, e), corresponding to C=O, C–O, and –OH groups^39^. Notably, the C–O peak area in KBC increases, while the C=O peak area decreases, indicating that high-temperature activation promotes oxidation, leading to the formation of additional C–O groups^40^. Furthermore, the FT-IR spectra of KBC reveal a weakening or complete disappearance of the C=O peak, further corroborating this transformation^41^. As is seen from Fig. 3c, the peaks of BC at 398.5 eV and 400.3 eV were pyridinic nitrogen and pyrrolic nitrogen, at high activation temperatures both formed a transition into graphitic nitrogen as illustrated in Fig. 3f. The higher electronegativity and reduced atomic radius of graphitic nitrogen markedly augmented the energy levels at defect sites as well as the charge density surrounding neighboring carbon atoms. This enhancement significantly promoted electron transfer within KBC, thereby facilitating both coordination and hydrogen bonding between the KBC surface and RhB, which in turn amplified adsorption efficacy^42,43^. Such phenomena were further substantiated through subsequent absorption experiments.

### RhB removal study

#### Effect of solution pH and dosage

**Fig. 4:**
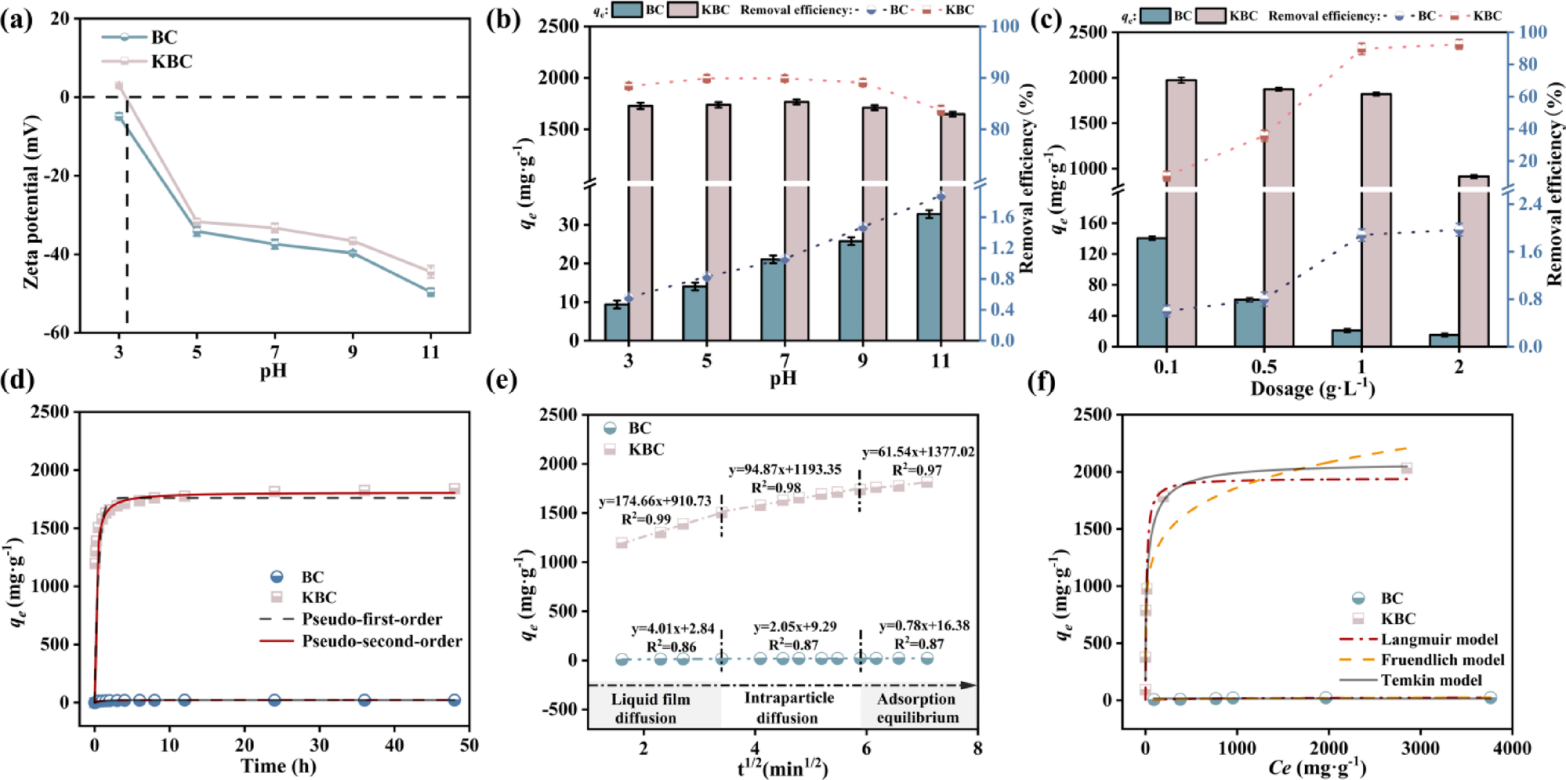
(**a**) Zeta potential, (**b**) pH, and (**c**) dosage on the adsorption of RhB, (**d**) adsorption kinetic model, (**e**) intraparticle diffusion model, and (**f**) isothermal adsorption model of RhB by BC and KBC.

The pH of the solution affected the state of oxygen-containing functional groups on the material’s surface and the chemical structure of the dye molecules. As shown in Fig. 4a, the Zeta potential of BC was observed to fluctuate between − 4.97 mV and − 49.72 mV, indicating that BC possessed a negative surface charge across a wide pH range. RhB, which is characterized as a positively charged dye, predominantly exists in its protonated form in aqueous environments, especially under acidic to neutral conditions. Under acidic conditions (pH = 3), the carboxylic acid groups (–COOH^−^) in RhB remain undissociated, while the amino groups are protonated to form –NH⁺, resulting in a cationic form of RhB^44^. At this pH, the Zeta potential is -5.30 mV, indicating a weak negative charge, and the high concentration of H⁺ in the solution competes with RhB for adsorption sites. The electrostatic repulsion between cationic RhB and the protonated BC surface further inhibits adsorption, leading to a low adsorption capacity of only 9.37 mg·g^−1^. With the pH gradually rising to 5 ~ 9, the Zeta potential range is − 35.61 ~ − 39.74 mV. During this range, RhB exists predominantly as a zwitterion, containing both anionic carboxyl (–COO^−^) and cationic amino (= N–^+^) groups^45^. BC surface electronegativity is enhanced, and can be combined with positive RhB through electrostatic attraction to enhance the adsorption capacity^21^.

For KBC, the point of Zero charge (pH_pzc_) was determined to be 3.18. Below this pH, the KBC surface was positively charged, while above it, the surface became negatively charged. When pH < 3.18, KBC is also positively charged, which would normally result in electrostatic repulsion with cationic RhB. However, adsorption may still occur due to strong hydrogen bonding and π–π interactions. The protonation or deprotonation of oxygen-containing functional groups is triggered by the binding of RhB, leading to increased oxygen exposure and facilitating hydrogen bond formation. Strong hydrogen bonding was found to enhance the KBC capacity for RhB in acidic environments^46^. As shown in Fig. 4b, in the range of pH 5 ~ 9, the Zeta potential is − 31.70 ~ − 36.72 mV, and the adsorption effect of KBC on RhB changes little and has no obvious rule. Thus, pH had minimal influence on adsorption here, indicating that electrostatic interactions were not dominant. It has been demonstrated by Li et al. that biochar with a graphitized exterior possesses substantial π-electron density, enabling it to function as a π-electron donor^47^. The KBC with a graphite structure functioned as a π-electron donor, whereas RhB was regarded as a π-electron acceptor, this enabled KBC to harness π–π electron donor–acceptor (EDA) interactions effectively to facilitate the adsorption of RhB into the pores. At pH values above 5, RhB may also convert to its hydroxide form, which further reduces its interaction with the active sites on KBC, leading to a slight decline in adsorption capacity^48^. Under the condition of pH > 9, the Zeta potential at this time is − 44.52 mV, and the electrostatic repulsion between KBC and RhB inhibits the adsorption in this process, resulting in a decrease in the removal rate^49^. Therefore, it appeared that the adsorption of RhB by KBC was largely independent of pH because π–π EDA interactions remained largely unaffected by pH changes. To ensure broader applicability in experiments, a neutral pH of 7.0 was selected for subsequent studies.

The dosage significantly impacted the adsorption process. As illustrated in Fig. 4c, the amounts of BC and KBC directly affected RhB adsorption. When the concentration levels were ramped up from 0.1 g·L^−1^ to 2 g·L^−1^, the capacity for adsorption within the samples took a nosedive, plummeting from a high of 1973.52 mg·g^−1^ and 140.52 mg·g^−1^ down to 919.99 mg·g^−1^ and 15.22 mg·g^−1^, respectively. Concurrently, the efficiency of the removal process saw a dramatic upswing, jumping from a meager 10.68% and a paltry 0.60% to an impressive 92.55% and a more substantial 2.97%, respectively. The increase in adsorption sites with higher dosages enhanced RhB removal efficiency as more interaction sites became available. However, overlapped active sites at the solid–liquid boundary decreased the net flow, potentially lowering the total adsorption capacity for BC and KBC^50^. Considering the cost implications of adsorbent dosage in pollutant removal, 1 g·L^−1^was selected as the optimal dosage for BC and KBC in subsequent studies, balancing efficiency, cost-effectiveness, and consistency. Under these conditions, BC adsorbed RhB at 23.42 mg·g^−1^ with an elimination efficiency of 1.88%, whereas KBC achieved an adsorption capacity of 1820.47 mg·g^−1^ and a removal rate of 89.77%.

#### Adsorption kinetics

The interaction kinetics between RhB and biochar were studied by fitting the pseudo-first-order, pseudo-second-order, and intra-particle diffusion models. Detailed fitting parameters and indices are provided in Table S3. It can be observed that the pseudo-first-order and pseudo-second-order kinetic models of BC exhibit high correlation coefficients. The pseudo-second-order model for KBC shows an excellent correlation (R^2^ > 0.98), with its equilibrium adsorption capacity being closer to the experimentally determined equilibrium adsorption (*q*_*e*_). This suggests that chemical interactions play a significant role throughout the adsorption process, which is quasi-irreversible^51^. Furthermore, the pseudo-first-order model for KBC also shows a good fit (Fig. 4d), indicating that physical adsorption, such as pore filling, plays a supplementary role in the adsorption process^52^. This finding suggests that adsorption capacity is proportional to the number of available active sites. KBC possesses a large specific surface area and a well-developed pore structure, providing more and more accessible active sites for RhB adsorption, which is one of the reasons for its excellent adsorption capacity.

The fitting results for the intraparticle diffusion of BC and KBC are shown in Table S4 and Fig. 4e. The adsorption process involves liquid film diffusion (Stage I), intraparticle diffusion (Stage II), and the adsorption equilibrium stage (Stage III)^53^, with the third stage not considered a rate-limiting step. Therefore, the rate-limiting step of the adsorption process is either intraparticle diffusion, liquid film diffusion, or a combination of both^54^. The k_di_ value of KBC is significantly higher than that of BC, indicating that KOH activation enables faster adsorption of RhB. Prior to reaching equilibrium, the small molecular structure of KBC facilitates the transport of RhB from the solution to the external surface of KBC via molecular and film diffusion, after which it enters the internal pores and is absorbed by the internal pores of KBC under the control of intraparticle diffusion. In contrast, BC adsorbs only through its external surface. Additionally, none of the fitting lines pass through the origin, suggesting that both liquid film diffusion and intra-particle diffusion limit the adsorption rate^55^.

#### Adsorption isotherms

Adsorption isotherm data for RhB were evaluated using Langmuir and Freundlich models, as summarized in Table S5. Figure 4f shows an upward trend in the initial concentration to correspond with a progressive enhancement in the adsorption capacity of RhB onto KBC and BC, which was driven by a greater pressure gradient that enhanced collisions between adsorption sites and RhB molecules, thereby improving the overall adsorption capacity^56^. As shown in Table S5, comparable fitting outcomes were obtained from the Langmuir and Freundlich isotherm models, both of which were found to describe the adsorption behavior of KBC effectively. The uptake of RhB by KBC encompassed both single molecule and heterogeneous multilayer adsorption. In the Freundlich model, *n*/1 reflected the difficulty of adsorption, values between 0.1 and 0.5 indicated easy uptake (with lower values signifying better performance) whereas values above 2 suggested difficulties in absorption. In this study, favorable conditions were observed for KBC (*n/1* = 0.16), which exhibited a stronger affinity for RhB compared to BC (*n/1* = 0.32). The Temkin model was also found to show a good fit with KBC, and its fitting coefficient *R*^2^ was 0.97, indicating that there were independent interactions among the adsorption sites, and π–π EDA interaction and ion exchange existed in the adsorption mechanism.

#### Adsorption thermodynamics

Upon rising from 298 to 308 K, the Δ*G*_θ_ for RhB adsorption onto BC and KBC stayed in the negative range, suggesting a spontaneous reaction (Table S6). The magnitude of Δ*G*_θ_ rose as temperature increased, indicating an enhanced tendency towards spontaneity at elevated temperatures. For KBC, Δ*G*_θ_ < –40 kJ·mol^−1^ and Δ*H*_θ_ < 25 kJ·mol^−1^ indicated that the adsorption was controlled by both physical adsorption and chemisorption^57^, consistent with kinetic results. In contrast, for BC, Δ*G*_θ_ > –20 kJ·mol^−1^ and Δ*H*_θ_ < 25 kJ·mol^−1^ suggested that the process was mainly governed by physical mechanisms. Moreover, compared with BC, KBC showed a lower Δ*G*_θ_ for RhB adsorption, proving that its efficiency improved as higher temperatures enhanced the movement of RhB molecules in solution. This resulted in more frequent contact with KBC and facilitated easier adsorption. In addition, thermal expansion may expose more adsorption sites. Furthermore, Δ*S*_θ_ > 0 for KBC compared with BC indicated an increase in freedom at the two-phase interface between KBC and RhB solution, further promoting adsorption.

#### Adsorption mechanism

**Fig. 5:**
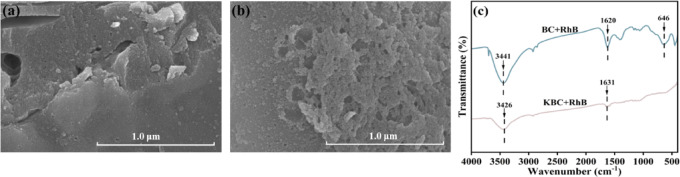
SEM image of (**a**) BC and (**b**) KBC, (**c**) FT-IR spectra after RhB sorption.

The comparative analysis of the physicochemical characteristics of the samples, both pre-and post-RhB adsorption, was conducted via SEM, FT-IR, and XPS techniques to uncover the underlying adsorption dynamics of RhB onto BC and KBC. The SEM imagery indicated that the surface texture and porosity of the samples sustained minimal alterations following adsorption (Fig. 5a, b). In contrast to BC, KBC was observed to exhibit fewer cracks and a smoother surface. This improvement was attributed to the reaction between RhB and KBC, where RhB was found to fill existing surface cracks and pore structures. Thus, pore filling was identified as a significant contributor to RhB removal by KBC. Furthermore, it was also demonstrated that the adsorption process did not appear to impact the structural integrity of either material, thereby highlighting their remarkable stability and enduring persistence^58^.

The FT-IR spectral analysis in Fig. 5c revealed a noticeable enhancement in the –OH stretching peak at 3421 cm^−1^ for both BC and KBC, attributed to hydrogen bonding between RhB and biochar^59^. After adsorption, the distinct peak observed at 1636 cm^−1^ was shifted, with BC experiencing a 16 cm^−1^ displacement and KBC shifting by 5 cm^−1^. This indicates that the C=C/C=O bonds participated in a chemical reaction with RhB, in conjunction with π–π EDA interactions occurring between the benzene ring of RhB and the C=C bonds present in the biochar^60,61^. Following RhB adsorption on BC, a new distinct C–H peak was observed at 646 cm^−1^, suggesting that the molecular structures of BC and RhB were more likely adsorbed through electrostatic or hydrogen bonding^43^.

**Fig. 6:**
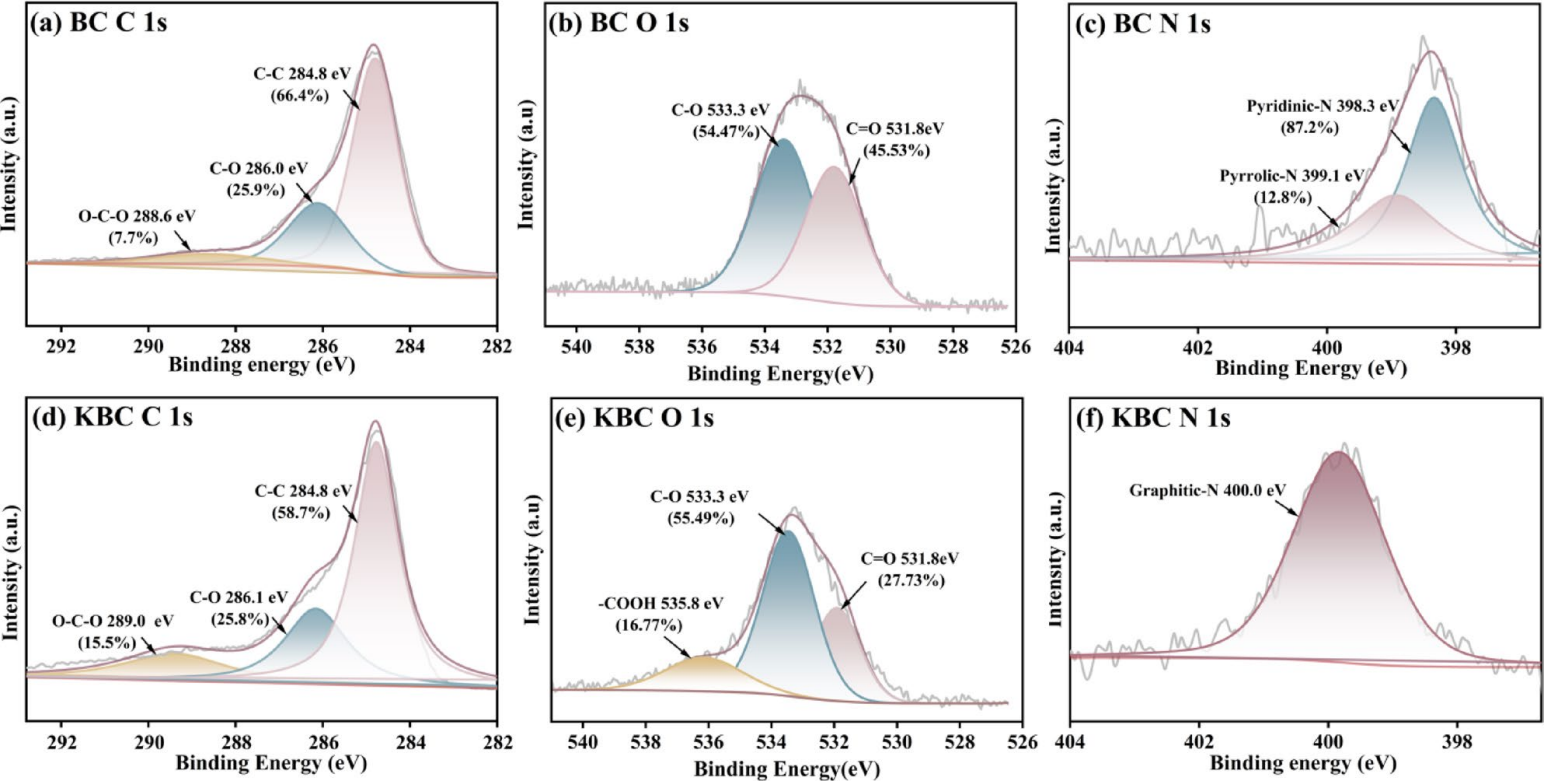
(**a**, **d**) XPS C 1 s, (**b**, **e**) O 1 s, and (**c**, **f**) N 1 s spectra for BC and KBC after adsorption of RhB.

**Fig. 7:**
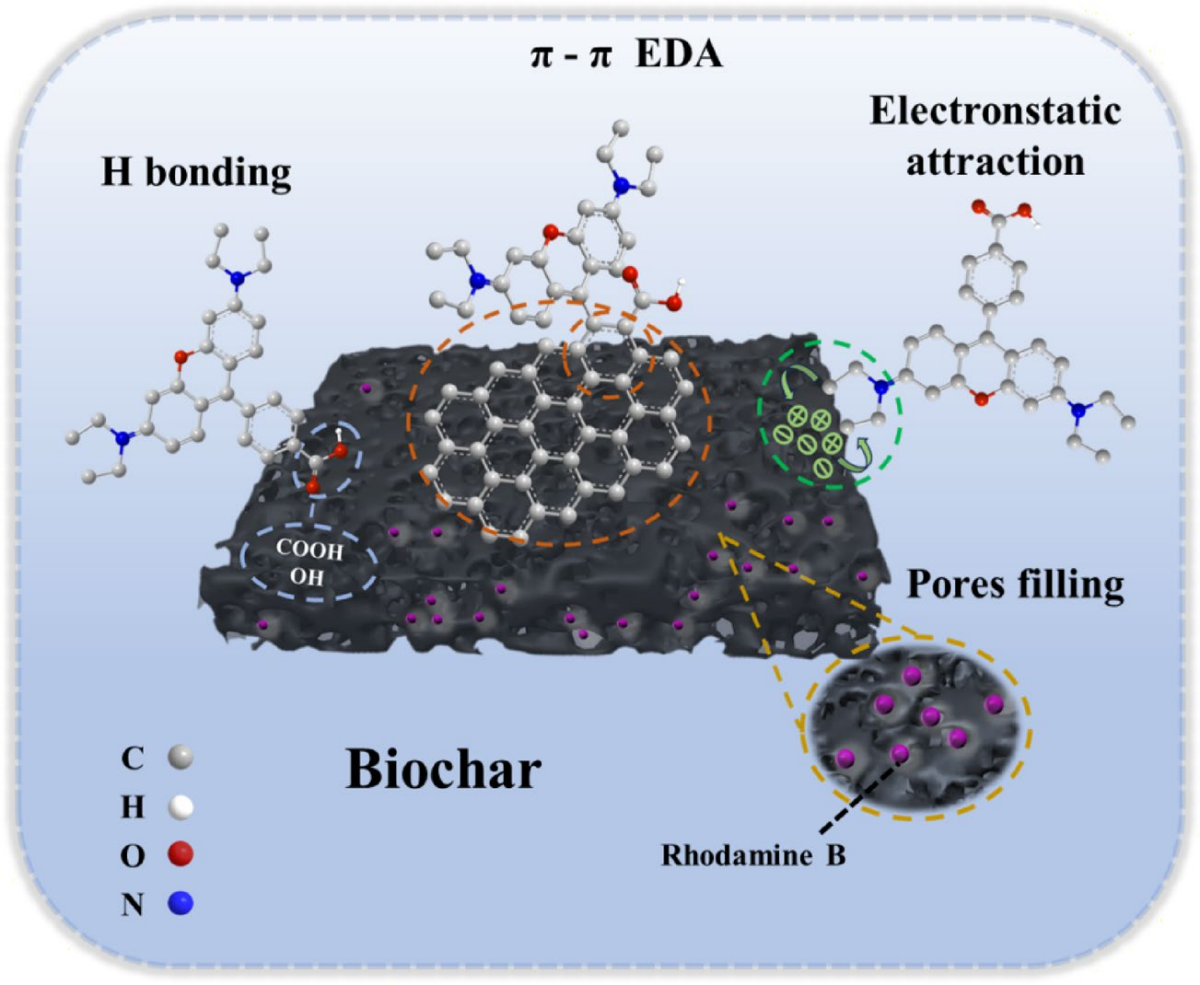
Adsorption mechanism of Rh B by KBC.

Upon examining the XPS images in Fig. 6, a comparison of the elemental composition and the types and peak areas of C, O, and N functional groups revealed notable differences before and after adsorption. Particularly, alterations were observed in the regions associated with C–C and C–O bonds within BC and KBC samples (Fig. 6a,d). The functional groups on the RhB molecule increased the characteristic peak areas of both, confirming that C–C and C–O participated in π–π EDA interactions for RhB removal. A similar trend was observed in the O 1 s spectrum (Fig. 6b,e), where the area of C–O for BC increased from 8.34 to 54.47% and for KBC from 21.15% to 55.49% post-adsorption. This was attributed to the low electron density and small amount of positive charge of the C–O group, which acted as an electron donor within the mechanism of EDA interactions^38^. In addition, the appearance of –COOH groups on KBC suggested the presence of adsorbed RhB on its surface. Hydrogen bonds were formed between the KBC –OH and –COOH groups and the RhB hydroxyl moieties. Furthermore, the –COOH groups were found to function as electron acceptors, facilitating RhB removal through π–π EDA interactions with aromatic structures^62,63^. Simultaneously, the surface of RhB was characterized by π-electron donor groups, which enhanced the electron density with the N atom in KBC and promoted the transfer of binding energy following C–H or N–H adsorption^64^. Figure 7 summarizes these potential interactions between the KBC and the RhB dye.

### Comparison with other adsorbents

The data in Table S7 reveal a comparison of the adsorption efficacy of biochar derived from various precursors on RhB. A striking observation is that the biochar produced through a KOH two-step activation process in our research outperformed the majority of previously documented adsorbents, boasting enhanced SSA and adsorption capacity. Unlike other adsorbents with complex preparation processes and high energy consumption, KBC operated under a wide range of environmental conditions, and KBC was an excellent adsorbent that was beneficial to the purification of dye wastewater.

### Cost–benefit analysis of KBC

Biochar materials necessitate careful consideration of engineering and economic viability for large-scale production. Cost estimates are influenced by raw material sourcing and energy consumption factors and do not include labor costs. Therefore, the techno-economic assessment of KBC was conducted based on the cost of chemical modifiers, the energy consumption during the pyrolysis process, and the stability of its removal performance. As shown in Table S8, the total cost of producing 1 g of KBC is USD 2.41, and the cost to remove 1 g of RhB from aqueous solution is USD 1.32. KBC demonstrates a clear cost advantage in RhB removal when compared with other adsorbents listed in Table S9, including activated carbon, biochar, and modified biochar. This demonstrates that converting *Spartina alterniflora* into biochar adsorbents represents a “win–win” strategy that can enhance waste management practices, reduce environmental pollution, and promote sustainable resource utilization.

## Conclusion

By performing a simple pyrolysis activation on the invasive plant *Spartina alterniflora*, a highly efficient and sustainable adsorbent was successfully synthesized. At a pyrolysis temperature of 450 °C, BC exhibited good adsorption performance, with an adsorption capacity of 23.42 mg·g^−1^. To enhance its performance further, KOH was used as an activator for modification. As the activation temperature increased, the adsorption capacity of KBC for RhB increased progressively. At an activation temperature of 800 °C, KOH activation significantly improved the adsorption capacity of KBC for RhB. KBC demonstrated excellent adsorption capabilities across a wide pH range, though the adsorption capacity decreased with increasing RhB concentration. Under conditions of 298 K and pH 7, 1 g·L^−1^ of KBC achieved a maximum adsorption capacity of 1820.47 mg·g^−1^ for 2000 mg·L^−1^ RhB, with a removal rate of 89.77%, which was 77.73 times higher than the original BC. Adsorption kinetics and isotherm modeling indicated that KBC’s adsorption of RhB followed a pseudo-second-order kinetic model and the Freundlich adsorption model, suggesting that the process was dominated by irreversible chemical reactions. Thermodynamic analysis revealed that the adsorption process was spontaneous and endothermic. Compared to BC, KBC exhibited a larger specific surface area (3109.67 m^2^·g^−1^) and a more developed pore structure (1.61 cm^3^·g^−1^), both of which contributed to its improved adsorption of RhB. The primary adsorption mechanisms of KBC for RhB included pore filling and π-π electron donor–acceptor (EDA) interactions, as well as hydrogen bonding and electrostatic interactions. These findings help to further elucidate the adsorption process and mechanisms of RhB on *Spartina alterniflora* biochar and provide valuable insights for the development of biochar-based adsorbents for dye removal in wastewater treatment, contributing to environmental pollution control efforts.

## Electronic supplementary material

Below is the link to the electronic supplementary material.

Supplementary Material 1

## Data Availability

Data is provided within the manuscript or supplementary information files.
