# Supplementary material for: Sustainable optimization of high specific surface area Spartina alterniflora biochar for Rhodamine B removal and mechanism
Source: Sci Rep. 2025 Jul 1;15:21745. doi: 10.1038/s41598-025-05714-6 (PMC12216906; doi:10.1038/s41598-025-05714-6)
Supplement: Supplementary file 1 — Supplementary Material 1 [file 41598_2025_5714_MOESM1_ESM.docx]

**Supplemental Information**

**Sustainable optimization of high specific surface area *Spartina alterniflora* biochar for Rhodamine B removal and mechanism**

Weiling Yu ^1, #^, Zhouyun Xie ^1, 2, #^, Ni Zhang ^1, 2^, Li Tang ^1^, Jingfen Xia ^1^, Jien Ye ^1, *^, Xuran Liu ^2^, Dongbo Wang ^2^, Guojing Yang ^1, *^

^1^College of Biological and Environmental Sciences, Zhejiang Wanli University, Ningbo 315100, P.R. China.

^2^College of Environmental Science and Engineering, Hunan University and Key Laboratory of Environmental Biology and Pollution Control (Hunan University), Ministry of Education, Changsha 410082, P.R. China.

*Corresponding authors. E-mail addresses: yejien@zwu.edu.cn (J. Ye), guojing_yang@163.com (G. Yang).

#The authors contributed equally.

**Text**

**Text S1:** Serial characterization of biochar

The specific surface area(SSA) and pore volume of biochar were determined by specific surface area (*S*_BET_) and pore analyzer (BET, Micromeritics, USA). The surface morphology of biochar was observed using a scanning electron microscope (SEM, Quanta FEI, USA). The material element information was determined by X-ray energy dispersive spectroscopy (EDS, S-4800X, Japan). The functional group type of biochar was determined using a Fourier transform infrared spectrometer (FT-IR, Nicolet IS10, USA). The phase analysis of biochar was carried out with an X-ray diffractometer (XRD, D8 Advance X, Germany). The element types and contents of biochar were determined using an X-ray photoelectron spectrometer (XPS, Thermo Scientific Escalab 250 Xi, USA). The electronegativity was analyzed using a Zeta potential analyzer (Zetasizer Nano ZS, UK).

**Figure**





**Fig. S1.** Performance comparison between BC and KBC at different pyrolysis temperatures.

**Tables**

| Materials | Yield (%) |
| --- | --- |
| BC450 | 45.15 |
| KBC600 | 33.43 |
| KBC700 | 29.13 |
| KBC800 | 25.85 |
| KBC900 | 11.19 |

**Table S1.** Yield of biochar materials after pyrolysis.

| Model | Equation | Constant |
| --- | --- | --- |
| ***Adsorption Kinetic*** |  | |
| Pseudo-first order | $\text{q}_{\text{t}}\text{=}\text{q}_{\text{e}}\text{（}\text{1-}\text{e}^{\text{-}\text{k}_{\text{1}}\text{t}}\text{）}$ | *k_1_*: adsorption rate constant |
| Pseudo-second order | $\text{q}_{\text{t}}\text{=}\frac{\text{k}_{\text{2}}\text{q}_{\text{e}}^{\text{2}}\text{t}}{\text{1+}\text{k}_{\text{2}}\text{q}_{\text{e}}\text{t}}$ | *k_2_*: adsorption rate constant |
| intraparticle diffusion | $\text{q}_{\text{t}}\text{=}k_{pi}\text{t}^{\text{1/2}}\text{+}\text{c}$ | *k_pi_*: adsorption rate constant;  *c*: boundary layer constant |
| ***Adsorption isotherm*** |  | |
| Langmuir | $\text{q}_{\text{e}}\text{=}\frac{\text{q}_{\text{max}}\text{K}_{\text{L}}\text{C}_{\text{e}}}{\text{1+}\text{K}_{\text{L}}\text{C}_{\text{e}}}$ | *K_L_*: Langmuir adsorption coefficient |
| Freundlich | $\text{q}_{\text{e}}\text{=}K_{F}{C_{e}}^{\frac{1}{n}}$ | *K_F_:* Freundlich constant |
| Temkin | $\text{q}_{\text{e}}\text{=}\frac{\text{RT}}{\text{b}}\text{ln}\text{K}_{\text{T}}\text{+}\frac{\text{RT}}{\text{b}}\text{ln}\text{c}_{\text{e}}$ | *K_T_*: evaluation binding constant;  *b*: constant; R: gas constant |
| ***Adsorption thermodynamics*** | $\text{lnK}\text{=}\frac{\text{Δ}\text{H}^{\text{θ}}}{\text{RT}}\text{+}\frac{\text{Δ}\text{S}^{\text{θ}}}{\text{R}}$ | $\text{Δ}\text{H}^{\text{θ}}$: enthalpy change;  $\text{Δ}\text{S}^{\text{θ}}$: entropy change |
|  | $\text{Δ}\text{G}^{\text{θ}}\text{=Δ-TΔ}\text{S}^{\text{θ}}$ | $\text{Δ}\text{G}^{\text{θ}}$: Gibbs free energy change |

**Table S2.** Equation and constant for adsorption kinetics, isotherm, and thermodynamics models.

| Biochar | Pseudo-first-order kinetics | | | Pseudo-second-order kinetics | | |
| --- | --- | --- | --- | --- | --- | --- |
|  | *q*_e_(mg·g^-1^) | *K*_1_(h^-1^) | *R*^2^ | *q*_e_(mg·g^-1^) | *K*_2_(g(mg·h)^-1^) | *R*^2^ |
| BC | 21.39 | 0.25 | 0.98 | 21.95 | 0.01461 | 0.98 |
| KBC | 1,699.69 | 0.18 | 0.93 | 1,749.87 | 0.00018 | 0.99 |

**Table S3.** Adsorption kinetic parameters of biochar for RhB obtained by pseudo-first-order and pseudo-second-order models.

| Biochar | *k_d1_*(mg·g^-1^·min^1/2^) | *C*(mg·g^-1^) | *R*^2^ | *k_d2_*(mg·g^-1^·min^1/2^) | *C*(mg·g^-1^) | *R*^2^ | *k_d3_*(mg·g^-1^·min^1/2^) | *C*(mg·g^-1^) | *R*^2^ |
| --- | --- | --- | --- | --- | --- | --- | --- | --- | --- |
| BC | 4.01 | 2.84 | 0.86 | 2.05 | 9.29 | 0.87 | 0.78 | 16.38 | 0.87 |
| KBC | 174.65 | 910.73 | 0.99 | 94.87 | 1,193.35 | 0.98 | 61.54 | 1,377.02 | 0.97 |

**Table S4.** Adsorption kinetic parameters of biochar for RhB obtained by intraparticle diffusion model.

| Biochar | Langmuir Isotherm Model | | | Freundlich Isotherm Model | | | Temkin Isotherm Model | | |
| --- | --- | --- | --- | --- | --- | --- | --- | --- | --- |
|  | *K*_L_(L·mg^-1^) | *q*_m_(mg·g^-1^) | *R*^2^ | *K*_F_ (g·(mg^(1-n)^·L^n^·g^-1^)) | *n* | *R*^2^ | *k_1_*(L·mg^-1^) | *k_t_*(J·mol^-1^) | *R*^2^ |
| BC | 0.002 | 28.14 | 0.88 | 1.953 | 3.17 | 0.83 | 5.23 | -17.81 | 0.82 |
| KBC | 0.09 | 1,945.04 | 0.92 | 606.57 | 6.16 | 0.90 | 178.42 | 630.99 | 0.97 |

**Table S5.** Adsorption constants of RhB with biochar from Langmuir、Freundlich and Temkin isotherm models.

| Biochar | T(K) | K(L·mol^-1^) | ΔG_θ_(kJ·mol^-1^) | ΔH_θ_(kJ·mol^-1^) | ΔS_θ_(kJ·mol^-1^) |
| --- | --- | --- | --- | --- | --- |
| BC | 298 | 0.0121 | -11.72 | -0.73 | 2.49 |
|  | 303 | 0.0174 | -24.19 |  |  |
|  | 308 | 0.0217 | -36.66 |  |  |
| KBC | 298 | 9.1118 | -4.74 | 7.86 | 42.28 |
|  | 303 | 9.6745 | -4.95 |  |  |
|  | 308 | 10.0996 | -5.16 |  |  |

**Table S6.** Adsorption constants of RhB with biochar from thermodynamic parameters.

| Adsorbent | Specific surface area (m^2^·g^-1^) | Dosage  (g·L^-1^) | Initial concentration  (mg·L^-1^) | *Q*_e_ (mg·g^-1^) | Literature Source |
| --- | --- | --- | --- | --- | --- |
| *Spartina alterniflora* biochar | 3,109.67 | 1 | 2000 | 1,820.47 | This work |
| Cassava residue biochar | 3.17 | 4 | 100 | 105.60 | [1] |
| Coconut Shell Biochar | 972.87 | 20 | 120 | 12.41 | [2] |
| Bamboo shoot biochar | 513.00 | 1 | 100 | 85.80 | [3] |
| Aquatic Nutmeg Biochar | 937.10 | 0.5 | 200 | 174.50 | [4] |
| Calophyllum inophyllum biochar | 155.39 | 1.2 | 200 | 190.00 | [5] |
| Luffa Biochar | 2,526.40 | 0.1 | 200 | 1,701.70 | [6] |
| Soybean cake biochar | 2,413.00 | 0.1 | 1000 | 839.97 | [7] |
| Algal bloom biochar | 2,169.00 | 0.1 | 80 | 1,101.00 | [8] |
| Peanut Shell Biochar | 351.11 | 0.325 | 100 | 508.25 | [9] |

**Table S7.** Comparison of biochar prepared from different raw materials and their adsorption performance for RhB.

|  | Prepare raw materials and prices | | | | Yield (%) | Remo-val (%) | Removal 1g RhB/ USD |
| --- | --- | --- | --- | --- | --- | --- | --- |
|  | materials | Unit/USD | Dosage | Cost/USD | 25.85 | 89.77 | 1.32 |
| KBC | *Spartina alterni-flora* | / | / | / |  |  |  |
|  | KOH | 2.88×10^-5^ g | 10 g | 2.88×10^-4^ |  |  |  |
|  | HCl | 1.5×10^-4^ g | 33.12 mL | 5.9×10^-3^ |  |  |  |
|  | N_2_ | 1×10^-3^ L | 0.96 L | 9.6×10^-4^ |  |  |  |
|  | Electricity | 6×10^-2^ Kwh | 40 Kwh | 2.4 |  |  |  |
|  | Total | / | / | 2.41 |  |  |  |

**Table S8.** Prepare cost and benefit of KBC.

| Materials | Cost/ USD | Literature Source |
| --- | --- | --- |
| Activated carbon | 2.5 | [10] |
| Biochar | 1.6~7.5 | [11] |
| Modified biochar | 2.59~9.5 | [12] |
| KBC | 2.41 | This work |

**Table S9.** Cost comparison between KBC and various adsorbent materials.

**References**

1. Wu, J., Yang J.W., Huang G.H., Xu C.H., Lin B.F. Hydrothermal carbonization synthesis of cassava slag biochar with excellent adsorption performance for Rhodamine B. J. Clean. Prod. **251**, 119717. <https://doi.org/10.1016/j.jclepro.2019.119717>(2020).
2. Li X.M., Shi J.X., Luo X.X. Enhanced adsorption of rhodamine B from water by Fe-N co-modified biochar: Preparation, performance, mechanism and reusability. Bioresour. Technol. **343**, 126103. <https://doi.org/10.1016/j.biortech.2021.126103> (2022).
3. Hou Y.R. *et al*. Hydrothermal conversion of bamboo shoot shell to biochar: Preliminary studies of adsorption equilibrium and kinetics for rhodamine B removal, J. Anal. Appl. Pyrolysis. **143**, 104694. <https://doi.org/10.1016/j.jaap.2019.104694> (2019).
4. Zhang D. *et al*. Preferential, synergistic sorption and reduction of Cr(VI) from chromium-rhodamine B mixed wastewater by magnetic porous biochar derived from wasted Myriophyllum aquaticum biomass. Environ. Pollut. **327,** 121593. <https://doi.org/10.1016/j.envpol.2023.121593> (2023).
5. Behera A.K., Shadangi K.P., Sarangi P.K. Efficient removal of Rhodamine B dye using biochar as an adsorbent: Study the performance, kinetics, thermodynamics, adsorption isotherms and its reusability. Chemosphere **354**, 141702. <https://doi.org/10.1016/j.chemosphere.2024.141702> (2024).
6. Su Y.J., Zheng Y.Y., Feng M.Q., Chen S.J. Magnetic Luffa-Leaf-Derived Hierarchical Porous Biochar for Efficient Removal of Rhodamine B and Tetracycline Hydrochloride. Int. J. Mol. Sci. **23**(24) 15703. <https://doi.org/10.3390/ijms232415703> (2022).
7. Zhang X.Y. *et al*. Highly-efficient nitrogen self-doped biochar for versatile dyes’ removal prepared from soybean cake via a simple dual-templating approach and associated thermodynamics. J. Clean. Prod. **332**, 130069. <https://doi.org/10.1016/j.jclepro.2021.130069> (2022).
8. Wang Y.S. *et al*. Insights into the highly efficient treatment of dyeing wastewater using algal bloom derived activated carbon with wide-range adaptability to solution pH and temperature. Bioresour. Technol. **349**, 126883. <https://doi.org/10.1016/j.biortech.2022.126883> (2022).
9. Kayranli B. *et al*. Peanut shell biochar for Rhodamine B removal: Efficiency, desorption, and reusability. Chemosphere **364**, 143056. <https://doi.org/10.1016/j.chemosphere.2024.143056> (2024).
10. Alhashimi H.A, Aktas C.B. Life cycle environmental and economic performance of biochar compared with activated carbon: A meta-analysis. Resour Conserv Recy. **118**, 13-16. <https://doi.org/10.1016/j.resconrec.2016.11.016> (2017).
11. Wijeyawardana P. *et al*. Assessing the life cycle and economic impact of cement-modified biochar compared to conventional adsorbents for heavy metal removal in stormwater. Process Saf Environ. **192**, 244-256. <https://doi.org/10.1016/j.psep.2024.10.050> (2024).
12. Chen M. *et al*. Low-cost Ca/Mg co-modified biochar for effective phosphorus recovery: Adsorption mechanisms, resourceful utilization, and life cycle assessment. Chem Eng J. **502**, 157993. <https://doi.org/10.1016/j.cej.2024.1579932024> (2024).
